# When the biological clock is disordered: circadian rhythm disruption as a core pathophysiological mechanism in anxiety and depression

**DOI:** 10.3389/fpsyt.2026.1842599

**Published:** 2026-08-05

**Authors:** Jiao Yuan, Jing Zhang, Menghui Yu, Lu Li

**Affiliations:** 1 Department of Psychology, Ward 1, Qingdao Mental Health Center, Qingdao, China; 2 Department of Sleep Medicine, Ward 4, Qingdao Mental Health Center, Qingdao, China; 3 Department of Sleep Medicine, Ward 3, Qingdao Mental Health Center, Qingdao, China

**Keywords:** anxiety, biomarker, chronotherapy, circadian rhythm, circadian rhythm disruption, depression

## Abstract

Anxiety and depressive disorders impose a heavy global burden. Although sleep disturbances have traditionally been regarded as secondary symptoms of these conditions, converging evidence now implicates circadian rhythm disruption as a core pathophysiological mechanism rather than a mere epiphenomenon. This review systematically explores the circadian system and constructs an integrated framework linking disruption to the synergistic dysregulation of multiple pathways and subsequent emotional/sleep abnormalities. We systematically examine five key mechanistic pathways: the SCN-limbic circuit, HPA axis, immune inflammation, neurotransmitter plasticity, and the gut-brain axis. Furthermore, we define actionable subtypes of disruption, summarize chronotherapies (e.g., light therapy, melatonin), and analyze current controversies regarding causality. Finally, we propose future directions, including multi-omics sampling and personalized intervention algorithms. By integrating evidence linking circadian disruption to anxiety/depression, this paper highlights its central mechanistic value, providing theoretical support for precision psychiatry and the integration of circadian medicine.

## 1 Introduction

Why circadian disruption should be elevated to a core mechanism.

Anxiety and depressive disorders are among the most common mental disorders worldwide, with a continuously increasing disease burden (1). Traditional perspectives regard sleep disturbances merely as concomitant symptoms of mood disorders (2). However, clinical practice and basic research suggest that merely extending sleep duration frequently fails to ameliorate emotional symptoms (3). This has prompted the academic community to re-evaluate the central role of “rhythm” rather than “sleep duration” in emotional regulation (4, 5). Recent evidence indicates that the circadian rhythm system, acting as the body’s primary endogenous timekeeping system, regulates functional oscillations across multiple systems, including the nervous, endocrine, and immune systems (6). The association between circadian disruption and mood disorders has evolved from descriptive correlation studies toward investigations of potential causal mechanisms. While circadian disruption may contribute to the onset and maintenance of mood disorders, current evidence-especially from human studies-remains limited, with modest effect sizes and ongoing challenges in establishing causal relationships (7, 8). This paper addresses three key questions: (1) How does circadian misalignment drive anxiety and depression through verifiable biological pathways? (2) Which symptom clusters correspond to different circadian phenotypes? (3) Which indicators are suitable for subtyping, prediction, and efficacy evaluation, and for which chronotherapies is the evidence strongest?

### 1.1 Definition of core concepts

#### 1.1.1 Biological clock

This represents the core timekeeping system maintaining circadian rhythms in the body, characterized by a “three-level regulatory network”. The molecular circadian clock, operating through transcriptional-translational feedback loops (TTFLs), constitutes the fundamental unit of rhythm regulation (9). Centered on the CLOCK/BMAL1-PER/CRY TTFL and modulated by auxiliary loops such as REV-ERB and ROR, it regulates the expression of downstream target genes and exists ubiquitously in cells throughout the body (10, 11). The neural clock utilizes the suprachiasmatic nucleus (SCN) as the central master clock (12, 13). Receiving light signals from retinal photoreceptors, it coordinates systemic rhythms via neural projections and humoral regulation, acting as the “conductor-in-chief” of the body’s rhythms. Peripheral clocks are distributed in peripheral tissues such as the liver, gut, and immune cells (14). While regulated by the SCN, they can also oscillate autonomously in response to zeitgebers such as feeding and exercise, thereby forming a synergistic “central-peripheral” rhythm network (14).

#### 1.1.2 Circadian disruption

This refers to structural and functional abnormalities of the circadian rhythm system, primarily manifested across five dimensions: (1) Phase abnormalities (delayed/advanced phase syndromes); (2) Amplitude abnormalities (reduced amplitude of rhythm oscillation and attenuated day-night differences); (3) Stability abnormalities (irregular rhythm fluctuations and high fragmentation); (4) Internal desynchrony (misalignment between the central and peripheral clocks, or among peripheral clocks); and (5) Environment-society time mismatch (misalignment between endogenous rhythms and external light, social, or eating times, such as shift work and social jetlag) (15–18). These disruptions do not exist in isolation; rather, they often occur in composite forms, collectively driving pathophysiological changes (19).

To ensure terminological clarity, we distinguish three related but distinct concepts used throughout this review: (1) Circadian disruption is used as the umbrella term referring to any structural or functional abnormality of the circadian rhythm system, encompassing phase, amplitude, stability, and synchrony abnormalities. (2) Circadian desynchronization specifically denotes internal misalignment among different circadian oscillators (e.g., between the central SCN clock and peripheral clocks, or among different peripheral clocks). (3) Circadian misalignment specifically denotes the mismatch between endogenous circadian rhythms and external environmental or social time cues (e.g., shift work, social jetlag). Throughout this review, we use “circadian disruption” as the primary term, reserving “desynchronization” and “misalignment” for contexts where the specific subtype of disruption is relevant.

#### 1.1.3 Distinction and association between sleep issues and rhythm issues

Sleep is a core physiological process regulated by the circadian system; however, rhythm issues encompass far more than quantitative abnormalities regarding “sleep duration” (20, 21). Their essence is a functional dysfunction in the “temporal dimension, “ including phase misalignment of the sleep-wake cycle, rhythmic shifts in peak hormone secretion (e.g., delayed melatonin secretion, blunted or absent morning cortisol peak), and the loss of oscillatory synchrony in neuroimmune functions (22–24). For instance, with an identical sleep duration of eight hours, a delayed phase pattern characterized by sleeping and waking late exhibits significant differences in cortisol rhythms and activity in emotion-regulating brain regions compared to a regular schedule pattern. This suggests that the “temporal characteristics” of the rhythm reflect emotional risk more accurately than sleep duration (25). Sleep problems can be viewed as one of the external manifestations of circadian disruption, while circadian disruption serves as the common pathological basis for both sleep abnormalities and mood disorders (26–28).

### 1.2 Preliminary evidence for causal possibility

Substantial clinical and basic evidence supports a causal association between circadian disruption and anxiety/depression, rather than a mere comorbid relationship (29–31). At the population level, natural experiments and epidemiological studies indicate that the incidence of anxiety and depression among shift workers (particularly those working night shifts or engaging in long-term transmeridian travel) is 20% to 40% higher than that of the general population (32). Furthermore, this association exhibits a dose-response relationship. Longitudinal studies of groups newly entering shift work have found that exposure to circadian misalignment precedes the onset of emotional symptoms, effectively ruling out reverse causation. In adolescent and college student populations, social jetlag (a sleep phase difference between weekdays and weekends of ≥ 2 hours) is significantly correlated with elevated depressive symptoms and anxiety vigilance (29, 33). A meta-analysis reveals that for every one-hour increase in social jetlag, the risk of depression increases by 11% (34).

At the level of basic research, animal experiments involving active manipulation of the circadian system have directly confirmed the causal role of circadian disruption in driving emotional states. Optogenetic activation or inhibition of SCN neurons significantly alters anxiety-like behaviors (elevated plus maze test) and depression-like behaviors (forced swim test, sucrose preference test) in mice (35). Light phase shift models (simulating jet lag) can induce core depressive symptoms such as anhedonia and social withdrawal in rats, with symptom severity positively correlating with the duration of circadian misalignment (36). Clock gene knockout mice (e.g., Per1/Per2 double knockouts) spontaneously exhibit anxiety- and depression-like behaviors, accompanied by disruptions in the rhythms of the HPA axis and monoamine neurotransmitter systems (37–39). This suggests that dysfunction of the molecular clock is an intrinsic trigger for mood disorders. Taken together, the evidence presented above establishes a causal chain linking circadian disruption to mood disorders across three levels of analysis—epidemiological, experimental, and molecular—thereby supporting the conceptualization of circadian disruption as a core mechanism underlying anxiety and depression.

## 2 Overview of the biological clock system: from molecular clock to neural circuit clocks

### 2.1 The molecular clock: the CLOCK/BMAL1-PER/CRY feedback loop and oscillatory output

The core of the molecular clock is the conserved transcription-translation feedback loop (TTFL), which generates a circadian oscillation of approximately 24 hours through periodic gene expression and protein degradation (40, 41). Specifically, CLOCK and BMAL1 form a heterodimer that binds to E-box elements in the promoters of *Per* and *Cry* genes, promoting their transcriptional expression. PER and CRY proteins accumulate in the cytoplasm to form complexes, subsequently translocate into the nucleus to inhibit the transcriptional activity of the CLOCK/BMAL1 heterodimer, leading to decreased expression of *Per* and *Cry* genes (42). Following the ubiquitin-dependent degradation of PER/CRY proteins, the inhibition is relieved, initiating a new round of transcription and establishing stable rhythmic oscillations. REV-ERBα/β and RORα/β further fine-tune the rhythm by regulating *Bmal1* expression (41, 43). Notably, the molecular clock does not merely function as a “timekeeper”; it also directly regulates metabolism (e.g., NAD+ synthesis), immune function (e.g., rhythmic expression of IL-6 and TNF-α), and neurotransmitter synthesis (e.g., circadian expression of synthetic enzymes for 5-HT and DA) (44). Polymorphisms in clock genes are associated with susceptibility to mood disorders; for instance, variations in the *Clock* gene are linked to bipolar disorder, and *Bmal1* is associated with seasonal depression (45–47).

Both clinical and basic evidence suggest that dysfunction of the molecular clock is closely related to anxiety and depression (48). Human genetic studies have found that the rs1801260 polymorphism in the *CLOCK* gene is significantly associated with susceptibility to depression (49); individuals carrying the mutant allele exhibit an increased incidence of depression accompanied by disrupted melatonin rhythms. Additionally, the methylation level of the *Per2* gene promoter is significantly elevated in the peripheral blood of patients with depression, leading to decreased PER2 protein expression and a reduced amplitude of molecular clock oscillations (50). In animal experiments, *Bmal1* knockout mice exhibit not only significant circadian disruption but also marked anxiety-like behaviors and anhedonia; supplementation with exogenous BMAL1 can reverse these emotional symptoms and molecular rhythm abnormalities (51).

### 2.2 The central master clock and multiple local brain clocks: “time organizers” of emotional circuits

The suprachiasmatic nucleus (SCN), located in the anterior hypothalamus, receives light signals via the retinohypothalamic tract and serves as the “master clock” coordinating peripheral clocks throughout the body (52). The SCN coordinates systemic rhythms via two core pathways: the neural projection pathway, which projects directly to emotion-related brain regions such as the hypothalamic paraventricular nucleus (PVN), amygdala, and prefrontal cortex (PFC) to regulate the rhythmicity of their neuronal activity (53, 54); and the humoral regulation pathway, which regulates peripheral tissue rhythms and hormone secretion (e.g., melatonin and cortisol) through the release of neuropeptides like arginine vasopressin (AVP) (54, 55). Accumulating evidence has demonstrated that key emotion-processing regions—the amygdala, PFC, nucleus accumbens (NAc), and hippocampus—each harbor independent local clocks (56, 57). These local clocks are regulated by the SCN but can also respond to local microenvironmental signals (e.g., neurotransmitters and inflammatory factors), forming a synergistic “central master clock-local brain clock” network (58).

Dysfunction of local clocks is closely related to symptom fluctuations in mood disorders (59). For example, the local clock in the amygdala regulates the formation and extinction of fear memories; desynchronization between this clock and the SCN can lead to over-consolidation of fear memories, manifesting as persistent anxiety vigilance. The local clock in the PFC regulates the circadian rhythm of emotional regulation functions; abnormalities in its oscillation can lead to rumination and decreased emotional regulation capacity, which aligns closely with the core symptoms of depression (60). The hippocampal local clock modulates synaptic plasticity through rhythmic expression of brain-derived neurotrophic factor (BDNF); its impairment can result in learning and memory deficits as well as anhedonia (19, 61). Furthermore, causal evidence from animal experiments further confirms that dysfunction of the SCN and local clocks is a significant contributor to mood disorders (24, 62).

## 3 The evidence lineage linking “circadian disruption to anxiety/depression”: from correlation to causation

### 3.1 Epidemiology and natural experiments: shift work, jet lag, and social jetlag

Shift work serves as a prototypical model of circadian misalignment. Longitudinal studies indicate a 28% increased risk of depression in shift workers (ES = 1.28), with a higher risk observed in females (32). Longitudinal changes in “new entrants to shift work” are particularly noteworthy; those with poor initial adaptation exhibit a subsequent significant increase in depressive symptoms, suggesting the role of individual differences in rhythm tolerance (63, 64). Social jetlag refers to the discrepancy in sleep midpoints between workdays and weekends (65). A meta-analysis reveals that social jetlag of ≥2 hours is significantly associated with depressive symptoms (OR = 1.44, 95% CI: 1.18-1.77), especially in adolescent and college student populations (29, 30). However, the effect size is small (r=0.16), suggesting that while circadian factors are a risk factor, they are not deterministic, and the modest effect sizes in human studies underscore the need for caution in attributing causal primacy to circadian disruption alone (32). Furthermore, population studies in extreme light environments (such as polar day or polar night regions) provide corroborating evidence for the pathogenicity of circadian disruption (66). Residents in polar night regions exhibit significantly higher rates of depressive symptoms compared to those in ordinary regions, accompanied by disrupted melatonin rhythms and low amplitude in activity rhythms (67). Supplementation with artificial light significantly improves both emotional symptoms and rhythm metrics in these populations, further supporting the causal link between circadian disruption and mood disorders (32).

### 3.2 Clinical phenotypes and biomarkers: phase, amplitude, melatonin, cortisol, and activity rhythms

Common circadian abnormalities in patients with depression or anxiety include: (1) Melatonin rhythm: Phase delay is common and is associated with symptoms such as anhedonia. (2) Diurnal cortisol profile: Hyperactivation of the HPA axis leads to an enhanced cortisol awakening response (CAR) and a flattened diurnal slope, which are associated with symptoms of anxiety vigilance. (3) Activity rhythm: Wrist actigraphy shows that patients with major depressive disorder exhibit reduced circadian amplitude (low CQ value), phase delay, and increased intradaily variability (IV). The CQ value is associated with the early response to antidepressant treatment (68, 69). As a “low-cost digital biomarker”, activity rhythm holds potential for clinical translation.

### 3.3 Animal and mechanistic causation: light phase shifts, rhythm deprivation, and clock gene models

**Figure 1:**
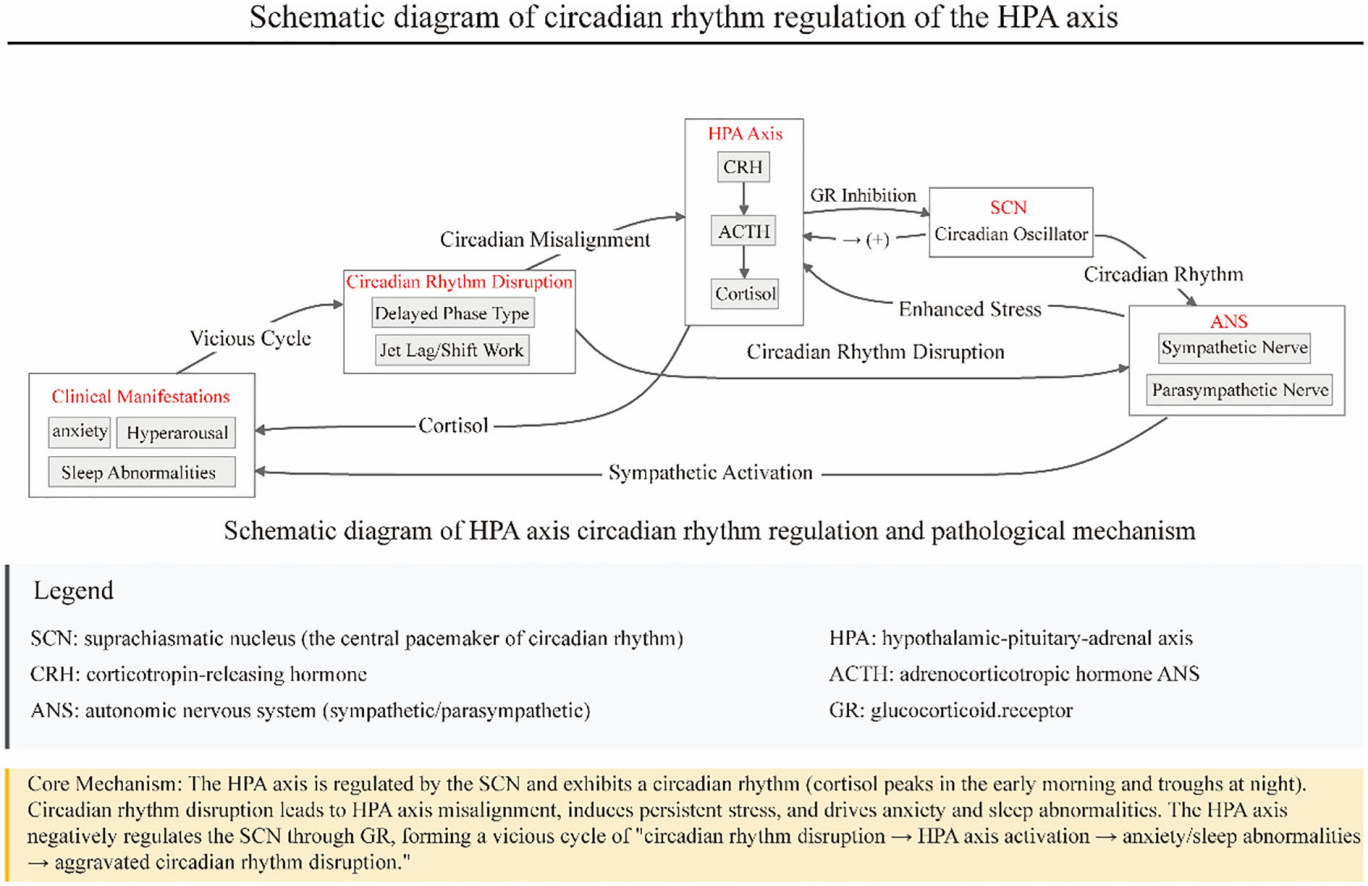
Schematic diagram of circadian rhythm regulation and pathological mechanism of the HPA axis.

Animal experiments provide direct causal evidence that circadian disruption drives anxiety- and depression-like phenotypes. By actively manipulating the circadian system, these studies have identified core regulatory pathways and furnished critical support for integrative mechanistic frameworks (70, 71). Animal models of light rhythm disturbance, such as repeated phase advances or constant light, can induce depression-like behaviors (reduced sucrose preference, prolonged immobility time in the forced swim test) and anxiety-like behaviors (reduced time spent in the open arms of the elevated plus maze), accompanied by reduced amplitude of molecular rhythms in the SCN and disruption of local clocks in the limbic system (72). SCN manipulation experiments further confirm the central role of the master clock; chemogenetic or optogenetic inhibition of SCN activity directly leads to increased anxiety-like behaviors, while SCN lesion models show disruptions in temperature and activity rhythms alongside abnormal emotional behaviors. Clock gene modified animal models confirm the pathogenicity of circadian disruption at the molecular level: knockout or mutation models of genes such as *Bmal1*, *Clock*, and *Per2* all display emotional behavioral abnormalities, accompanied by disruptions in the rhythms of the serotonin (5-HT) and dopamine (DA) neurotransmitter systems (73). Furthermore, different gene mutations correspond to distinct behavioral phenotypes (e.g., *Clock* mutations tend toward mania-like behavior, whereas *Bmal1* mutations tend toward depression-like behavior). Among the multiple pathophysiological pathways verified by animal experiments, the hypothalamic-pituitary-adrenal (HPA) axis is a core pathway linking circadian disruption to anxiety and sleep abnormalities (74). Its circadian regulatory process and pathological changes induced by rhythm disruption are highly specific, which are visually summarized in **Figure 1**.

### 3.4 Intervention evidence: chronotherapeutics improving mood

Intervention evidence is a critical link in verifying causal relationships (69, 75). Current research shows that chronotherapeutic interventions (light therapy, melatonin/agonists, phase advance, sleep deprivation plus phase advance, interpersonal and social rhythm therapy) can improve depressive symptoms, particularly in patients with circadian delay (76). Whether “rhythm realignment” constitutes one of the antidepressant mechanisms has become a core scientific question. Melatonin and receptor agonists (e.g., ramelteon) exert therapeutic effects by correcting phase abnormalities: for patients with delayed-phase depression, taking low-dose melatonin (0.5–1 mg) two hours before bedtime can significantly advance the dim light melatonin onset (DLMO), improving sleep phase and emotional symptoms (77). Ramelteon, as an MT1/MT2 receptor agonist, not only improves sleep but also alleviates anxiety and depressive symptoms by realigning molecular clock oscillations, with significantly fewer side effects than traditional anxiolytics (78). Behavioral rhythm interventions (e.g., interpersonal and social rhythm therapy, timed exercise/feeding) improve rhythm stability and internal synchrony by strengthening zeitgebers: interpersonal and social rhythm therapy can significantly reduce the risk of recurrence in patients with depression, while simultaneously improving activity rhythms and cortisol rhythms (79). Timed exercise (30 minutes of daily morning exercise) enhances rhythm amplitude and improves symptoms of anxiety vigilance, with efficacy comparable to that of light therapy (80).

## 4 Core pathophysiological mechanism modules

**Figure 2:**
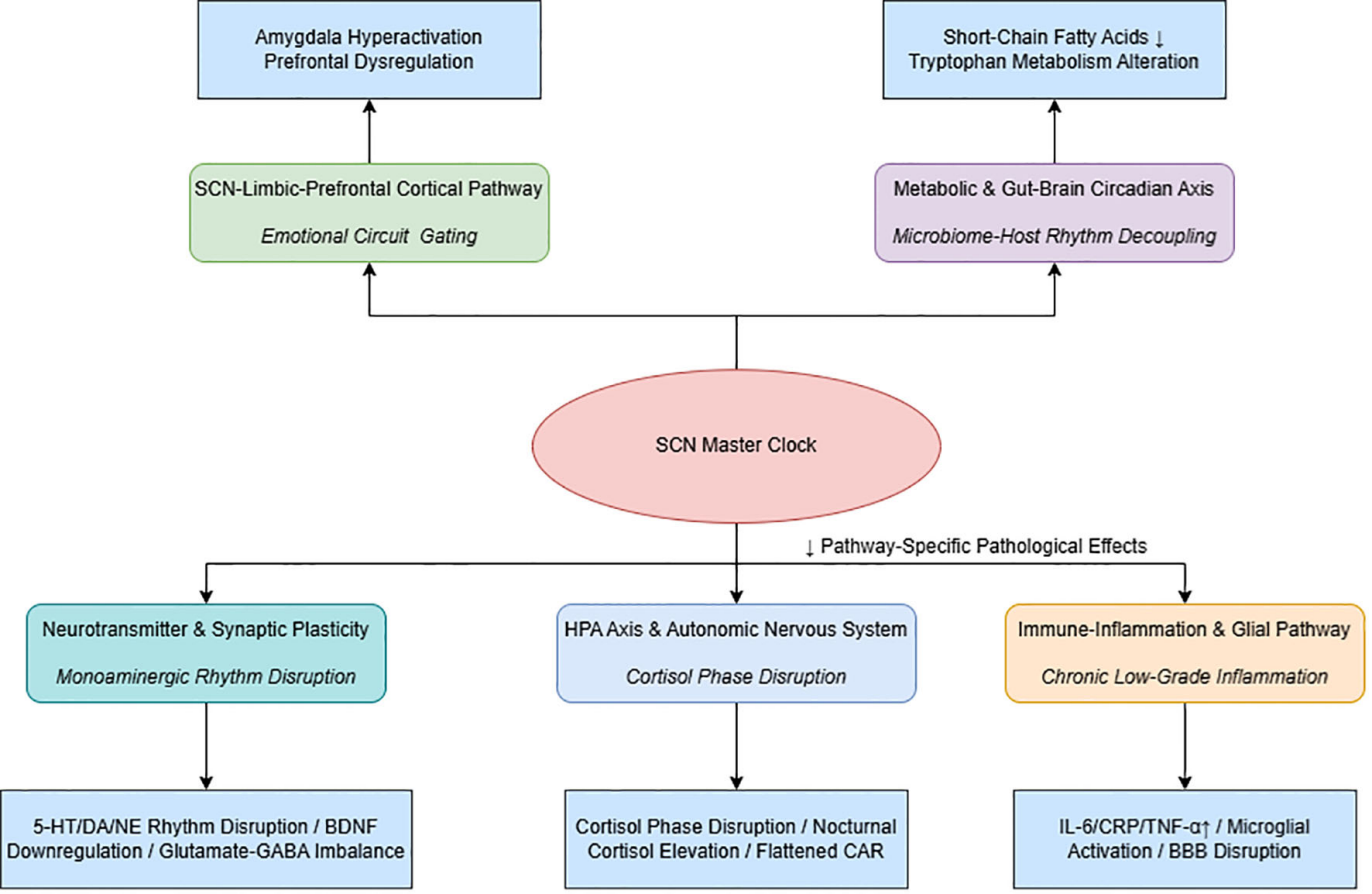
Schematic diagram of core pathophysiological mechanism modules.

The core mechanism by which circadian disruption drives anxiety and depression is not a single pathway; rather, it acts through the synergistic effects of five major pathways: the “SCN-limbic system circuit” (71), the “HPA axis” (81), “immune inflammation” (82), “neurotransmitters and synaptic plasticity” (83), and “metabolism and the gut-brain axis” (84). These interactions form a cascade reaction: “circadian desynchronization → multisystem dysfunction → emotional circuit imbalance.” Concurrently, symptoms of anxiety and depression can further disrupt the function of the circadian system, creating a vicious cycle. This section constructs a multi-pathway convergence model to systematically elucidate the regulatory mechanisms and interactions of each pathway (**Figure 2**).

The suprachiasmatic nucleus (SCN), the master circadian pacemaker, regulates downstream processes through core clock genes (CLOCK/BMAL1, PER/CRY). Circadian disruption propagates through five interconnected pathways: (1) the SCN–limbic–prefrontal cortical pathway (emotional circuit gating), (2) the HPA axis and autonomic nervous system (cortisol phase disruption), (3) the immune-inflammatory and glial pathway (chronic low-grade inflammation), (4) the neurotransmitter and synaptic plasticity pathway (monoaminergic rhythm disruption), and (5) the metabolic and gut–brain circadian axis (microbiome–host rhythm decoupling). Each pathway produces distinct pathological effects (bottom row) that converge on the clinical phenotype of anxiety and/or major depression. Color-coded boxes distinguish each pathway: green for the SCN-limbic-prefrontal pathway, purple for the metabolic and gut-brain axis, cyan for neurotransmitter and synaptic plasticity, blue for the HPA axis and autonomic nervous system, and orange for the immune-inflammatory and glial pathway. Abbreviations: SCN, suprachiasmatic nucleus; CAR, cortisol awakening response; CRP, C-reactive protein; TNF-α, tumor necrosis factor-alpha; IL-6, interleukin-6; 5-HT, serotonin; DA, dopamine; NE, norepinephrine; BDNF, brain-derived neurotrophic factor; BBB, blood-brain barrier.

### 4.1 SCN-limbic system-prefrontal cortex: failure of “time gating” in emotional circuits

The suprachiasmatic nucleus (SCN) orchestrates activity rhythms in key emotion-processing regions—including the amygdala, prefrontal cortex (PFC), and nucleus accumbens (NAc)—through direct neural projections, thereby establishing a “time-gating” mechanism that modulates emotional circuits. This mechanism regulates the diurnal fluctuations of functions including threat processing, reward processing, and emotion regulation (18, 85). Circadian disruption can lead to the failure of this gating function, triggering emotional abnormalities.

*Threat-processing pathway.* The amygdala, a central hub for threat detection, exhibits robust circadian rhythmicity: neuronal activity peaks in the early morning and reaches a trough during the night, a pattern that prevents excessive vigilance (86). Circadian misalignment (e.g., delayed phase, light disturbance) can weaken SCN regulation over the amygdala, causing desynchronization in the activity rhythm of amygdala neurons (68). This manifests as a persistently high activity state, leading to anxious vigilance and over-consolidation of fear memories, which aligns closely with the core symptoms of anxiety disorders. Animal experiments have confirmed that mice with impaired SCN-amygdala projection pathways spontaneously exhibit anxiety-like behaviors and significantly heightened reactivity to threat stimuli (87). In the reward processing pathway, the synergistic activity rhythms of the NAc and PFC regulate hedonic experiences and motivational behaviors (19). Circadian disruption can lead to abnormal rhythms in dopamine (DA) secretion in the NAc, while weakening PFC regulatory control over the NAc, resulting in anhedonia and reduced motivation—core pathological features of depression (88). In the emotion regulation pathway, the PFC maintains emotional homeostasis by inhibiting excessive activity in the amygdala. Circadian disruption can cause disorganization in PFC neuronal activity rhythms and weaken its inhibitory function, rendering it unable to effectively regulate amygdala activity, while also leading to rumination and decreased emotion regulation capacity (4).

### 4.2 HPA axis and autonomic nervous system: from “phase misalignment” to “hyperarousal-anxiety”

As visualized in **Figure 1**, the hypothalamic-pituitary-adrenal (HPA) axis maintains a stable circadian rhythm under normal physiological conditions, which is tightly regulated by the SCN (86). Circadian misalignment causes alterations in the phase of the cortisol peak (often delayed) and a flattening of the diurnal slope. Under normal circumstances, the morning cortisol peak provides an “alertness drive”; however, in individuals with phase delay, the peak occurs later in the morning, manifesting as exacerbated morning anxiety and difficulty maintaining sleep (82). Environmental-social time mismatches, such as shift work and social jetlag, can lead to continuous activation of the HPA axis, a flattened diurnal cortisol slope, and elevated nighttime cortisol levels (33, 34, 66). This triggers a state of chronic hyperarousal while simultaneously inhibiting sleep initiation, forming a vicious cycle of “circadian disruption → HPA activation → anxiety/sleep abnormalities.” Circadian disruption can also cause autonomic dysfunction, characterized by sustained sympathetic activation, manifested as reduced heart rate variability (HRV) and elevated blood pressure (89). This further enhances the stress response of the HPA axis, exacerbating symptoms of anxious vigilance. Notably, there is bidirectional regulation between the HPA axis and the SCN (35). HPA axis activation can inhibit SCN neuronal activity via glucocorticoid receptors (GR), disrupting circadian homeostasis. Concurrently, elevated peripheral glucocorticoid levels in patients with depression can inhibit circadian oscillations in the SCN, leading to aggravated circadian disruption.

### 4.3 Immune inflammation and neuroglia: rhythm is the master scheduler of immune oscillation

Immune cells (e.g., macrophages, T cells) and inflammatory factors (IL-1β, IL-6, TNF-α) all exhibit circadian rhythms regulated by clock genes. Circadian desynchronization leads to immune rhythm disruption → chronic low-grade inflammation → increased risk of depression-like behaviors (90). This pathway can be described as: circadian disruption → inflammatory signals (e.g., IL-6) crossing the blood-brain barrier → activation of microglia → influence on neurotransmitter metabolism and synaptic plasticity → emotional symptoms (91). Anti-inflammatory interventions (e.g., IL-6 receptor antagonists) have shown efficacy in a subset of depressed patients, supporting the inflammatory mechanism (92).

### 4.4 Neurotransmitters and plasticity: monoamines, glutamate/GABA, BDNF, and synaptic homeostasis

Circadian rhythms regulate the synthesis, release, and receptor sensitivity of monoamine neurotransmitters (5-HT, DA, NE) (44, 93). Desynchronization leads to functional disturbances in these neurotransmitter systems, corresponding to symptoms such as “anhedonia, cognitive slowing, and rumination”. The balance between glutamate and GABA is also regulated by circadian rhythms; NMDA receptor function and GABAergic inhibition both exhibit diurnal variations (93, 94). Synaptic plasticity (e.g., LTP/LTD) possesses specific circadian windows, and circadian disruption attenuates plasticity efficiency. Brain-derived neurotrophic factor (BDNF), as a key plasticity factor, has its expression regulated by clock genes. SCN dysfunction leads to aberrant activation of the BDNF-TrkB pathway in the striatum, mediating depressive and anxiety behaviors (19).

### 4.5 Metabolism and the gut-brain-circadian axis: an emerging integrated perspective

The gut-brain-circadian axis is a frontier field that has emerged in recent years (84). Feeding rhythms, gut microbial rhythms, and metabolite oscillations are coupled with host rhythms (84). Microbial composition and metabolic products (short-chain fatty acids, tryptophan metabolites) exhibit diurnal fluctuations and are regulated by host clock genes and feeding times. Circadian disruption (e.g., shift work, late-night eating) leads to reduced microbial diversity, loss of metabolite rhythms, HPA axis dysfunction, and enhanced neuroinflammation. Depressed patients commonly exhibit gut microbiota dysbiosis (e.g., reduction in *Faecalibacterium*, increase in *Enterobacteriaceae*), and microbe-host circadian desynchronization may be one of the mechanisms underlying treatment resistance (68). Time-restricted feeding (TRE), as a “chrono-nutrition” strategy, may improve mood by reconstructing microbial rhythms.

## 5 Clinical subtyping and measurable indicators: translating mechanisms into practice

### 5.1 Operational subtyping of circadian disruption

Based on existing evidence, it is recommended to categorize circadian disruption associated with mood disorders into at least four types: (1) Delayed Phase Type: Characterized by late sleep and wake times, poor morning function, and delayed dim light melatonin onset (DLMO), commonly observed in adolescent depression. (2) Low Amplitude/Fragmented Type: Characterized by reduced differences between day and night activity, fragmented sleep, and weak activity rhythms, associated with anhedonia and psychomotor retardation. (3) Internal Desynchronization Type: Characterized by a lack of synchronization among the sleep-wake rhythm, melatonin rhythm, and core body temperature rhythm, leading to significant symptom fluctuations. (4) Social/Environmental Misalignment Type: Primarily caused by exogenous factors such as shift work, social jetlag, and transmeridian travel (95).

### 5.2 Indicators and methods: the “toolkit”

The circadian assessment toolbox includes: (1) Questionnaires: Morningness-Eveningness Questionnaire (MEQ), Munich Chronotype Questionnaire (MCTQ), social jetlag calculation, and PSQI/ISI for assessing sleep quality. (2) Wearable Devices: Wrist actigraphy used to calculate non-parametric indicators such as Interdaily Stability (IS), Intradaily Variability (IV), and Circadian Quotient (CQ), serving as low-cost digital biomarkers. (3) Biological Markers: Dim Light Melatonin Onset (DLMO, the gold standard for phase), diurnal cortisol curve (morning Cortisol Awakening Response [CAR], diurnal slope), and core body temperature rhythm. (4) Frontier Directions: “Digital rhythm phenotyping”, which involves long-term tracking via smartphones and wearable devices combined with machine learning to identify individualized rhythm characteristics and patterns of symptom fluctuation (96, 97).

## 6 Intervention and precision chronotherapy

### 6.1 Light/dark therapy: core strategy for phase correction and amplitude enhancement

*Mechanism*: Light acts on the suprachiasmatic nucleus (SCN) via the retinohypothalamic tract to induce phase shifts—morning light produces phase advances, whereas evening light produces phase delays—and to amplify circadian amplitude.

*Target subtypes*: Delayed sleep-wake phase type, seasonal depression, and low-amplitude type.

*Evidence*: Light therapy has the strongest evidence base for seasonal affective disorder (SAD), with effect sizes of d = 0.8–1.2; its effect for non-seasonal depression is moderate (d = 0.3–0.5) (69).

*Implementation*: Morning light therapy: 10, 000 lux for 30–60 min, initiated immediately upon waking. Dark therapy: restrict blue-light exposure in the evening (e.g., blue-light-blocking glasses; see **Table 1**).

**Table 1:** Summary of circadian rhythm disorder subtypes and corresponding chronotherapies in mood disorders.

Circadian rhythm disorder subtypes	Core phenotypes	Assessment indicators	Preferred chronotherapies	Adjuvant interventions	Target population
Delayed Phase Type	Delayed sleep onset, difficulty waking up in the morning, and few nocturnal awakenings	Sleep diary, wearable devices, melatonin rhythm detection	Morning light therapy (7:00-9:00, 30 minutes per day) (Cohen’s d = 0.53 – 0.86 for depression, response rate ~50 - 60% (100, 101)	Low-dose melatonin before bedtime (0.5–1 mg)	Adolescents, night owls, and patients with depression accompanied by difficulty falling asleep
Advanced Phase Type	Early sleep onset, early awakening, daytime sleepiness, and frequent nocturnal awakenings	Sleep diary, core body temperature rhythm detection	Evening light therapy (18:00-20:00, 30 minutes per day) (Cohen’s d = 0.39-0.71 for depression; response rate ~45-55%) (100)	Regular daytime activities and avoidance of afternoon naps	Elderly people and patients with anxiety accompanied by early awakening
Low Amplitude Type	Small circadian rhythm fluctuations, fragmented sleep, and persistent low mood	Cortisol rhythm detection, clock gene expression detection	Regular light therapy combined with behavioral rhythm reinforcement (fixed schedule) (Cohen’s d = 0.42-0.65) (102)	Combined melatonin intervention and exercise intervention	Patients with severe depression and chronic insomnia complicated with anxiety
Internal Desynchronization Type	Misalignment between the central clock and peripheral clocks, disrupted sleep-wake rhythm, and emotional instability	Multi-time point peripheral clock gene expression detection, intestinal flora detection	Individualized light therapy combined with dietary rhythm adjustment	Probiotic intervention (regulating the gut-brain axis), clock gene modulators	Shift workers and people under chronic stress

### 6.2 Melatonin and rhythm pharmacology mechanism

*Mechanism*: Melatonin serves as the hormonal signal of the circadian clock. Acting primarily through MT1/MT2 receptors, exogenous melatonin induces phase shifts; administration in the afternoon or early evening produces phase advances (98). Agomelatine possesses a dual mechanism of action as an MT1/MT2 agonist and a 5-HT2C antagonist. *Target Subtypes*: Delayed phase type, internal desynchronization type. *Evidence*: Melatonin is effective for Delayed Sleep-Wake Phase Disorder (DSWPD) comorbid with depressive symptoms. Meta-analyses of agomelatine show efficacy comparable to SSRIs but with superior tolerability and more significant improvement in sleep architecture (99). *Implementation*: Low-dose melatonin (0.5–3 mg) taken 2–3 hours before bedtime; Agomelatine (25–50 mg) taken at bedtime (liver function monitoring required, **Table 1**).

### 6.3 Behavioral and lifestyle rhythm interventions mechanism

By regulating zeitgebers such as feeding, exercise, and social interactions, these interventions strengthen the synchrony between peripheral and central clocks, improve rhythm stability and internal synchrony, and break the vicious cycle linking circadian disruption to emotional abnormalities (83). Target Subtypes: Applicable to all types, especially social misalignment type and low amplitude type. Evidence: RCT studies have confirmed that Interpersonal and Social Rhythm Therapy (IPSRT) can significantly reduce the risk of recurrence in patients with depression (30% reduction in 1-year recurrence rate) while improving activity rhythms and cortisol rhythms. Daily moderate-intensity exercise for 30 minutes in the morning (e.g., brisk walking, jogging) can enhance rhythm amplitude and improve symptoms of anxious vigilance. Timed eating (fixed times for three daily meals, with an interval of ≥3 hours between dinner and sleep) can restore gut microbial rhythms and improve somatic symptoms and emotional states (18, 66, 69). Implementation: IPSRT requires the development of a personalized schedule, including fixed wake-up times, 30 minutes of outdoor light exposure in the morning, regular meal times (avoiding late-night eating and bingeing, while reducing intake of high-sugar and high-fat foods to support gut microbiota homeostasis), and timed exercise (**Table 1**) (84).

### 6.4 Intervention as a test of mechanism

If circadian realignment can alleviate depression, this constitutes compelling evidence supporting the hypothesis that circadian disruption is a core pathogenic mechanism rather than a mere epiphenomenon. A systematic review has indicated that patients with baseline phase delay derive the greatest benefit from chronotherapeutic interventions, underscoring the importance of subtype-matched treatment. Future research should employ rigorous RCT designs (e.g., chronotherapy versus non-chronotherapy active controls) to further validate the chronotherapeutic mechanism and to explore the synergistic effects of multimodal chronotherapy (light + melatonin + behavioral intervention). 7. Controversies and methodological pitfalls.

Although evidence for the causal relationship between circadian disruption and anxiety/depression continues to accumulate, research remains fraught with controversies and methodological pitfalls (19, 86). This paper addresses these issues from four key perspectives:

1. Causal Direction: Does depression cause circadian disruption first, or does circadian disruption precede depression? Most existing studies are cross-sectional, making it difficult to establish causality. Longitudinal studies suggest that sleep disturbances often precede depressive episodes; however, reverse causation may also occur, potentially creating a vicious cycle. Resolving this controversy requires long-term longitudinal studies and causal inference approaches (e.g., Mendelian randomization, natural experiments) to clarify the temporal sequence and causal direction between circadian disruption and mood disorders.
2. Confounding Factors: Variables such as sleep duration, physical activity, substance use, medications, and socioeconomic factors are all correlated with both circadian rhythms and mood. Some studies show that the association between social jetlag and depression weakens after controlling for sleep duration, suggesting the need to disentangle the independent effects of sleep and circadian rhythms.
3. Indicator Heterogeneity: Significant heterogeneity exists in the definitions and detection methods of circadian indicators, hindering cross-study comparisons. For example, multiple calculation methods exist for social jetlag (different formulas in the MCTQ questionnaire), and research studies employ varying thresholds (e.g., ≥1 hour vs. ≥2 hours). Future research must establish standardized protocols and thresholds for circadian indicator assessment to enhance result comparability.
4. Inconsistent Evidence: Some studies report that after adjusting for confounders, the association between circadian disruption and emotional symptoms weakens significantly or loses statistical significance. For instance, certain adult cohort studies show that the link between social jetlag and depressive symptoms disappears after adjusting for sleep quality; some animal experiments find that certain clock gene knockout mice do not exhibit obvious emotional abnormalities. This inconsistency may relate to population/animal heterogeneity, differences in intervention/perturbation methods, or variations in detection timing. It suggests that the association between circadian disruption and mood disorders may be condition-dependent. Future research should clarify the boundary conditions of this association (e.g., individual differences, gender differences, population differences).

## 7 Conclusion and future directions

This paper proposes an integrated framework: Environmental factors (light exposure, shift work, social jetlag) and intrinsic factors (clock gene polymorphisms, SCN function) jointly lead to circadian desynchronization (delayed phase/low amplitude/internal desynchronization). This, through multiple pathways including the SCN-limbic system circuit, HPA axis, immune inflammation, neurotransmitter/plasticity, and gut-brain-circadian axis, drives functional imbalance in emotional circuits, manifesting as symptom clusters of anxiety/depression. Meanwhile, sleep abnormalities and circadian disruption become intertwined, with symptoms themselves (e.g., nocturnal awakenings, daytime sleepiness) further disrupting rhythms and forming a vicious cycle (66, 84).

Future research directions:

1. Time-resolved multi-omics sampling: Combining transcriptomic, metabolomic, and microbiomic sampling at multiple time points to map individualized “circadian omics profiles”.
2. Causal inference: Utilizing Mendelian randomization and natural experiments (e.g., daylight saving time transitions) to strengthen the causal associations between circadian disruption, emotional symptoms, and sleep abnormalities.
3. Personalized algorithms: Developing personalized prescriptions for light/exercise interventions based on machine learning.
4. Stratified RCTs by circadian phenotypes: Validating chronotherapy efficacy by stratifying patients based on circadian phenotypes to advance precision psychiatry.

Incorporating circadian assessment and chronotherapeutic interventions into routine clinical practice holds promise for improving treatment outcomes for anxiety/depression, alleviating sleep disturbances, and reducing associated disease burdens. Circadian disruption represents a potentially pivotal mechanism—though not the only one—for mood disorders (depression, anxiety) and sleep abnormalities, and the evidence is strongest when circadian disruption is considered as a contributing factor rather than a sufficient cause (40, 69).
